# Supplementary material for: Non‐Hermitian Boundary in a Surface Selective Reconstructed Magnetic Weyl Semimetal
Source: Adv Mater. 2025 Feb 5;37(14):2419559. doi: 10.1002/adma.202419559 (PMC11983250; doi:10.1002/adma.202419559)
Supplement: Supplementary file 1 — Supporting Information [file ADMA-37-2419559-s001.pdf]

ADVANCED MATERIALS

# Supporting Information

for *Adv. Mater.*, DOI 10.1002/adma.202419559

Non-Hermitian Boundary in a Surface Selective Reconstructed Magnetic Weyl Semimetal

*Cong Li*, Yang Wang, Jianfeng Zhang, Guowei Liu, Hongxiong Liu, Wanyu Chen, Hanbin Deng, Wenbo Ma, Craig Polley, Balasubramanian Thiagarajan, Timur K. Kim, Jiaxin Yin, Youguo Shi, Tao Xiang* and Oscar Tjernberg**

Supplementary Material for

# Non-Hermitian Boundary in a Surface Selective Reconstructed Magnetic Weyl Semimetal

Cong Li[1,♯,*], Yang Wang[1,♯], Jianfeng Zhang[2,♯], Guowei Liu[3,♯], Hongxiong Liu[2,♯], Wanyu Chen[1], Hanbin Deng[3], Wenbo Ma[2], Craig Polley[4], Balasubramanian Thiagarajan[4], Timur K. Kim[5], Jiaxin Yin[3], Youguo Shi[2], Tao Xiang[2,*], Oscar Tjernberg[1,*]

[1]*Department of Applied Physics, KTH Royal Institute of Technology, Stockholm 11419, Sweden*

[2]*Beijing National Laboratory for Condensed Matter Physics, Institute of Physics, Chinese Academy of Sciences, Beijing 100190, China*

[3]*Department of Physics, Southern University of Science and Technology, Shenzhen, Guangdong 518055, China*

[4]*MAX IV Laboratory, Lund University, 22100 Lund, Sweden*

[5]*Diamond Light Source, Harwell Campus, Didcot, OX11 0DE, United Kingdom*

[♯]*These people contributed equally to the present work.*

[*]*Corresponding authors: conli@kth.se, txiang@iphy.ac.cn, oscar@kth.se*

## 1. Crystal characterizations and sample dependent measurements of NdAlSi

Figure S1a shows the results of energy dispersive x-ray (EDX) measurements on NdAlSi. The average composition determined by EDX is very close to the stoichiometric ratio of Nd:Al:Si = 1:1:1 (Fig. S1a). Fig. S1b shows the refined powder x-ray diffraction (XRD) patterns of NdAlSi at room temperature. The Bragg reflections can be well refined with reliability parameters, demonstrating high-quality NdAlSi crystallized into the non-centrosymmetric structure with a space group of $I4_1/md$ (no. 109). The refined lattice parameters are $a = b = 4.204$ $\AA$, $c = 14.519$ $\AA$, consistent with those reported in literature[1, 2]. The single crystal XRD pattern of NdAlSi is shown in Fig. S1c. Fig. S1d shows the temperature-dependent resistivity $\rho$(T) measured under zero field which exhibit two transition temperatures correspond to ~3.4 K and ~7.3 K respectively.

Figure S2 shows the partial results of NdAlSi measurements taken from 8 different samples, the terminal surface obtained after the cleavage of samples 1-4 corresponds to surface 1, terminal surface obtained after the cleavage of samples 5-8 corresponds to surface 2. Both Fermi surfaces are reproducible and are obtained in the manner shown in Fig. 1d-1f of the main text. The entirely asymmetrical surface states observed on the upper and lower surfaces further confirm the non-centrosymmetric structure of NdAlSi.

## 2. Fermi surface of NdAlSi measured under linear horizontal and linear vertical polarizations

Figure S3 shows the Fermi surfaces of NdAlSi measured on surface 1 (Fig. S3a-S3b) and surface 2 (Fig. S3c-S3d) with photon energy of 41 eV under linear horizontal (LH) and linear vertical (LV) polarizations. This provides further information on the electronic structure of different terminal surfaces in NdAlSi.

## 3. Constant energy contours comparison between measured and calculated results

In order to eliminate the possibility of band energy shift between the measured and calculated results, we did the constant energy contour (CEC) measurements with the photon energy of 41 eV under linear horizontal (LH, Fig. S4b) and linear vertical (LV, Fig. S4c) polarizations. Fig. S4a shows the surface projected density functional theory (DFT) CEC calculations of the Al terminal surface cleavage at the Al-Nd layer without considering the

surface reconstruction. It can be found that no matter how much energy is staggered, the measured results cannot be consistent with the surface projected DFT CEC calculations without considering the surface reconstruction. Therefore, the scenario of a Fermi level shift between measurements and calculations was excluded.

### 4. Twinned Fermi surface of NdAlSi

Figure S5a shows the lattice structure diagram of the Al atom cleavage plane. Through crystal structure analysis it is seen that there are two possible cleavage locations in the Al-Nd layer (C1 and C2 in Fig. S5a). If the sample is cleaved from C1 in Fig. S5a, the distribution of Al atoms on the cleaved surface is shown in Domain1 in Fig. S5a, while if the sample is cleaved from C2 in Fig. S5a, the distribution of Al atoms on the cleaved surface (Domain2, Fig. S5a) rotates 90 degrees with respect to Domain1 (Fig. S5a). Since the fracture locations of C1 and C2 are equivalent on the lattice, the two domains are equally likely to exist on the surface. The same is true for the Nd atoms on the cleaved surface at the Al-Nd layer. Therefore, if the light spot covers the two domains at the same time during the measurement, the measured Fermi surface is a superposition, as shown in Fig. S5b-S5c.

### 5. Terminal surface dependent Fermi surface calculations of NdAlSi

Figure S6a shows the crystal structure of NdAlSi, which crystallizes in the tetragonal structure with the space group $I4_1md$ (no. 109)[1]. From the perspective of crystal structure, there are three possible cleavage planes in NdAlSi. These are cleavage at the Si-Al layer, cleavage at the Al-Nd layer and cleavage at the Nd-Si layer, as shown in Fig. S6b. Therefore, there are potentially six different terminal surfaces in NdAlSi (Fig. S6g-S6i, Fig. S6m-S6o) corresponding to six different surface electronic structures. To visualize the difference between the six terminal surfaces, we performed surface-projected DFT band calculations along the $\overline{M}-\overline{\Gamma}-\overline{Y}(\overline{X})-\overline{M}-\overline{X}(\overline{Y})-\overline{\Gamma}$ directions on these six terminal surfaces, as shown in Fig. S6d-S6f and Fig. S6j-S6l. The surface projected DFT Fermi surface calculations on these six terminal surfaces are shown in Fig. S7. From the calculations, it can be noted that the electronic structures of different terminal surfaces are expected to differ substantially.

### 6. The surface projected phonon spectrum calculations of NdAlSi

Figure S8 shows the calculations of the projected phonon spectrum of the surface on the

surfaces terminated with Nd (Fig. S8a) and Al (Fig. S8b) without considering any surface reconstruction. The surface projected phonon spectrum exhibits negative frequencies on the Al (Fig. S8b) terminated surface, thus indicating an unstable surface. The Nd (Fig. S8a) terminated surface, on the other hand, shows no negative frequencies.

## 7. Comparison of the Fermi surfaces and constant energy contours between measured and calculated results on surface 2 of NdAlSi

Figures S9a and S9b show the Fermi surfaces and constant energy contours of NdAlSi measured on surface 2 with a photon energy of 41 eV under LV (Fig. S9a) and LH (Fig. S9b) polarizations, which visualizes the evolution of the electronic structures as a function of binding energy. After considering the 2×1 surface reconstruction on the Al terminated surface, the surface projected DFT Fermi surface and constant energy contours are shown in Fig. S9c. By comparison, it is found that the calculated constant energy contours are in good agreement with the measured after considering the 2×1 surface reconstruction on the Al terminated surface.

Figure S10 shows the Fermi surfaces of NdAlSi measured with a photon energy of 97 eV on surface 1 (Fig. S10a) and surface 2 (Fig. S10b) under LH polarization, which gives a larger momentum range view of the electronic structures.

## 8. Scanning tunneling microscopy measurements of NdAlSi

Figure S11 shows the scanning tunneling microscopy (STM) measurements on Al (Fig. S11a -S11c) and Nd (Fig. S11d-S11f) terminated surfaces. In the measurements, step-like structures can be observed at both the Al and Nd atomic planes. The line profile across the step shows that the step height measured in the Al plane is c/4, and the step height measured in the Nd plane is c/2. By analyzing the lattice structure, we find that the arrangement of atoms in the Al(Nd) plane rotates 90 degrees every c/4 intervals along the c direction (Fig. S11b, S11e). It is consistent with the STM measurements in Fig. S11a, S11d. It is seen that there is a 2×1 surface reconstruction on the Al terminated surface (Fig. S11a and Fig. S12) but not on the Nd-terminated surface (Fig. S11d). Fig. S11c and S11f show the larger scale of the Al (Fig. S11c) and Nd (Fig. S11f).

Such surface-selective spontaneous reconstruction observed in NdAlSi is a rare occurrence. Generally, after cleavage, either no reconstruction occurs, or both cleavage surfaces are

reconstructed. Then, we analyzed the chemical bonds in NdAlSi and found that Si-Al bonds are covalent, while Al-Nd and Nd-Si bonds are metallic. Covalent bonds are often directional, with specific bond angles and lengths compared to metallic bonds[3]. This directional nature can make covalent-bonded surfaces more prone to reconfiguration in response to external influences. The relatively soft nature of the metallic bonds allows surface stress after cleavage to relax on the Nd-terminated surface. For the Al-terminated surface, the surface stress causing the 2×1 surface reconstruction is relieved at the Nd layer below the Al and Si atomic layers, leaving the lower bulk atomic layers unaffected. Thus, surface reconstruction and relaxation occur only in the first one-quarter unit cell below the surface (Fig. 2k).

Locating large flat well defined surfaces on NdAlSi using STM is challenging. The main reason is that NdAlSi is a, hard to cleave, three-dimensional crystal. After cleavage, most regions on the surface of NdAlSi exhibit a stepped morphology (Fig. S12a), with relatively flat and well-defined areas being confined to small regions. Additionally, the effectiveness of STM in identifying flat, well-defined regions is significantly lower than that of ARPES since ARPES is a surface integrating technique whereas STM requires time consuming sequential scanning. STM data shown in Fig. S11c, S12b and S11f do, however, show that relatively large flat areas of Al and Nd terminated surfaces can be found using STM with sufficient time and effort.

### 9. Band structure calculations for surface 2 of NdAlSi, taking into consideration the surface reconstruction

Figure S13 shows the surface projected DFT calculated band dispersion along $\overline{M}-\overline{\Gamma}-\overline{Y}(\overline{X})-\overline{M}-\overline{X}(\overline{Y})-\overline{\Gamma}$ directions on the Al terminated surface, taking into account the 2×1 surface reconstruction. This shows the influence of surface reconstruction on the electronic structure in NdAlSi.

### 10. Photon energy dependent Fermi surface mapping on surface 2

Figure S14 shows the photon energy dependent Fermi surface mapping of NdAlSi measured on surface 2. The two arc features with strong spectral weight as marked by black arrows in Fig. 3b also observed in the Fermi surfaces measured with a photon energy of 34 eV (Fig. S14a) and 37 eV (Fig. S14b). Furthermore, the arc features on the Fermi surfaces (Fig. S14a-S14c) exhibit negligible photon energy dependence.

## 11. Confirmation of Fermi arcs

To distinguish whether the observed arc features in Fig. S15a-S15b are parts of a close contour, we did the band dispersions measurements on surface 2 of NdAlSi with photon energy of 41 eV along Cut1-Cut4 under PC polarization (Fig. S15c-S15f) and along Cut5-Cut8 under NC polarization (Fig. S15g-S15j). The surface Fermi arc (SFA) can be clearly observed along Cut1-Cut2 and Cut7-Cut8, and some blurred SFA signals along Cut 3 and Cut 6 but below the Fermi level. They completely disappears along Cut4 and Cut5. Some sharp band features (marked by orange arrows) can be observed near Fermi level along Cut 3 and Cut 6 but they are not derive from the arc features. Thus, we can confirm that the arc features observed in Fig. S15a-S15b are indeed arcs, and not the parts of a close contour.

In order to study the connectivity of the Fermi arcs, the circular polarization dependent measurements were made on surface 2, as shown in Fig. S16. Fig. S16a-S16b show the Fermi surface map of surface 2 measured with photon energy of 41 eV under PC (Fig. S16a) and NC (Fig. S16b) polarizations. The difference between PC and NC polarizations gives the circular-dichroic (CD) results of the Fermi surface. It can be found that the arc feature observed in Fig. 3b is a mixture of two different lengths of arcs. The mixture of two different lengths of arcs is further confirmed by the circular polarization dependent band structure measurements, as shown in Fig. S16d-S16r. Furthermore, it is note that the ends of both arcs of different lengths (SFA1 for longer one, SFA2 for shorter one) are very close to where the Weyl points of opposite chirality are located, as shown in Fig. S16c. Previous reports indicate that the actual Weyl point energy positions are slightly higher than the Fermi energy (38 meV higher for the inner pair and 56 meV higher for the outer pair of Weyl points)[5]. Considering the projection of the arc features, the ends of both arcs of different lengths should align exactly with the Weyl points of opposite chirality. Therefore, we confirm that the arc features in Fig. 3b are the SFAs.

Then, we further examine the signatures of chiral charge in NdAlSi based on the bulk boundary correspondence between the bulk Weyl fermions and surface Fermi arcs. In order to do so, we examine the signatures of chiral charge in the close loop cuts, as shown in Fig. S17. For a closed loop in the surface BZ where the bulk band structure is everywhere gaped we add up the signs of the Fermi velocities of all surface states along this loop, with Chern number n = +1 for right movers and n = -1 for left movers. Since SFA1 and SFA2

are very close each other, in order to reduce signal interference with each other, we did close loop band cuts analysis in circular polarization dependent measurements. Fig. S17c-S17f show the unrolling of the closed loop cuts along Loop 1 (Fig. S17c), Loop 2 (Fig. S17d), Loop 3 (Fig. S17e) and Loop 4 (Fig. S17f). Loop 1 and Loop 3 only shows a right-moving chiral mode while a left-moving chiral mode is observed for Loop 2 and Loop 4. The sum of all projected chiral charges along each loop cut gives the non zero Chern number[4], indicating the topological Fermi arcs nature of the observed arc features in Fig. S17a and Fig. S17b.

### 12. Diagram of Weyl-node connections on surface 2

Figure 3h shows the Fermi arc schematic on surface 2, taking into account the surface reconstruction. In order to clearly show how the nodes are connected we show a schematic of the arcs in Fig. S18. Fig. S18a and S18b show the Fermi arcs connecting the inner (Fig. S18a) and the outer (Fig. S18b) Weyl nodes. The longer SFAs (green one on the left and red one on the right in Fig. S18c) connect the outer pair of Weyl points and the shorter SFAs (red one on the left and green one on the right in Fig. S18c) connect the inner pair of Weyl points. The superposition is seen in Fig. S18c (Fig. 3h).

### 13. Comparison of surface states of surface 2 and bulk states in NdAlSi

Figure S19a shows the bulk state Fermi surface of NdAlSi with photon energy of 41 eV ($k_z \sim 0\ \pi/c$) under LH polarization. The DFT calculated bulk Fermi surface of NdAlSi at the $k_z = 0\ \pi/c$ plane, integrated $0 \pm 0.1\ \pi/c$ over the BZ along the $k_z$ direction is shown in Fig. S19b. The numerical calculation can be seen to capture most of the experimentally observed features.

Figure S20 shows the comparison of surface states of surface 2 and bulk states in NdAlSi. Fig. S20c-S20e show the band dispersions measured along Cut1-Cut3 (red lines in Fig. S20a). According to the previous analysis, the band marked by the orange arrow is SFA, which forms a hybrid state with the band marked by the green line. Further comparison between the band marked by the green line (Fig. S20c-S20e) and the measured bulk bands (Fig. S20f-S20h) shows that they have a good coincidence, especially in Cut3 (Fig. S20h). Therefore, it can be determined that there is a hybrid state of SFA and bulk state on the reconstructed surface of NdAlSi, and of course, there are also some contributions of conventional surface states and SFA hybridization in this hybrid state.

## 14. Quasiparticle scattering rate of SFA

To compare the quasiparticle lifetimes of surface Fermi arcs (SFA) on reconstructed and non-reconstructed surfaces in NdAlSi, we analyzed the quasiparticle scattering rates of SFA on surface 1 and surface 2, as shown in Fig. S21. Fig. S21a and S21d are the Fermi surface measured on Nd (non-reconstructed, Fig. S21a) and Al (reconstructed, Fig. S21d) terminated surface with photon energy of 41 eV under LH polarization. Fig. S21b and S21e show the measured band structure acrossing the SFAs on Nd (Cut1, Fig. S21b) and Al (Cut2, Fig. S21e) terminated surface. The SFAs are marked by black arrows. SFA shows significantly wider broadening on reconstructed surfaces (Fig. S21e) than on non-reconstructed surfaces (Fig. S21b). To further quantitatively analyze the scattering rate of terminated surface dependent SFA, we extracted the EDC passing through the corresponding SFA, as shown in Fig. S21c and S21f. The fitted EDC show a scattering rate of 18 meV for SFA1 and 52 meV for SFA2 on unreconstructed surface (Fig. S21c), and 210 meV for SFA on the reconstructed surface (Fig. S21f). The reciprocal of the quasiparticle scattering rate indicates the quasiparticle lifetime. Thus, the quasiparticle lifetime of an SFA on the non-reconstructed surface (Fig. S21c) is about 4 to 11 times longer than that on the reconstructed surface (Fig. S21f).

## 15. Discussion of non-Hermitian exceptional points and skin effects in artificial systems and surface-reconstructed Weyl semimetals

If one platform offers precise control over dissipation and coupling, non-Hermitian exceptional points (EPs) can be demonstrated by tuning parameters to achieve the coalescence of eigenvalues and eigenvectors. Systems such as optical setups, photonic crystals, or electronic circuits are particularly suitable for this purpose, as they enable controlled introduction of gain and loss, which is essential for realizing EPs.

For the non-Hermitian skin effect (NHSE), its observation requires the platform to support asymmetric coupling mechanisms and allow for both open and periodic boundary conditions. NHSE can then be identified by comparing eigenstate distributions under these different conditions. Breaking reciprocity is a key requirement for demonstrating NHSE, which can be achieved using asymmetric couplers or directional amplifiers in photonics, or unidirectional elements in circuits.

Demonstrating non-Hermitian EPs and NHSE in surface-reconstructed Weyl semimetals, however, presents unique challenges. Despite this, it remains a feasible endeavor. In the following, we will discuss the potential realization of non-Hermitian EPs and NHSE in surface-reconstructed Weyl semimetals separately.

The non-Hermitian exceptional points (EPs) are distinctive degeneracies in the spectrum of a non-Hermitian Hamiltonian, characterized by the coalescence of both eigenvalues and their associated eigenvectors. Unlike degeneracies in Hermitian systems, where eigenvectors remain orthogonal, EPs render the Hamiltonian defective, meaning it cannot be fully diagonalized due to the loss of linear independence among eigenvectors.

In the context of surface-reconstructed Weyl semimetals, if a newly generated non-topological surface Fermi arc (NTSFA) crosses with a topologically protected surface Fermi arc (SFA), the crossover point may display the properties of a non-Hermitian EP under certain conditions. This is because the crossover point of the SFA and NTSFA caused by the non-Hermitian effect guarantees eigenvalue coalescence at that point, so if the eigenvectors are coalescent at this point it will constitute an exceptional point. However, achieving eigenvector coalescence is a challenging open question, which requires a special model to be constructed in theory. Experimentally, there are observable criteria that could indicate the presence of an EP at the crossover point, such as whether the eigenvalues near the crossover point of the NTSFA and SFA exhibit square-root dispersion. Such experimental signatures provide a pathway for identifying and characterizing non-Hermitian EPs in these systems. According to our current measurements, there is no crossover between NTSFA and SFA in NdAlSi, so there are no EPs in NdAlSi.

The Non-Hermitian skin effect (NHSE) refers to a phenomenon in non-Hermitian systems where an extensive number of eigenstates accumulate at the boundaries (edges or surfaces) of the system. This effect arises due to the non-Hermitian nature of the Hamiltonian, which can break the conventional bulk-boundary correspondence observed in Hermitian systems.

Surface-reconstructed Weyl semimetals can be viewed as a Hermitian bulk – non-Hermitian boundary system. Since the bulk remains Hermitian and only the boundary exhibits non-Hermitian behavior, these systems do not exhibit the traditional non-Hermitian skin effect (NHSE), where a large number of bulk eigenstates accumulate at the boundaries. In a broader sense, however, the NHSE describes the boundary localization in non-Hermitian systems caused by non-reciprocal hopping or other asymmetries. Under this definition, a

surface NHSE can be considered present in surface-reconstructed Weyl semimetals.

As discussed in the main text, the formation of non-topological surface Fermi arcs (NTSFAs) originates from asymmetric surface electron hopping in momentum space, induced by surface reconstruction. Without this asymmetry, the spectral weight of the NTSFAs vanishes. The non-Hermitian effects of surface reconstruction in Weyl semimetals manifest in two primary ways:

(1) Generation of NTSFAs due to asymmetric surface electron hopping.

(2) Broadening of both SFAs and NTSFAs, stemming from non-Hermitian dissipation.

For topological electrons, the NTSFAs act as an effective open boundary condition (OBC). When these electrons traverse the reconstructed surface, some flow back into the bulk along the SFA, while others scatter onto the NTSFA, transforming into non-topological electrons. Unlike the bulk electrons, those scattered onto the NTSFA cannot form closed loops under an applied magnetic field. Consequently, these dissipative electrons accumulate on the reconstructed surface, eventually reaching a new equilibrium between electron accumulation and dissipation.

We refer to this accumulation of non-topological dissipative electrons on the reconstructed surface, driven by the non-Hermitian nature of the boundary, as the non-Hermitian boundary electron skin effect. This phenomenon can be interpreted as a special form of the NHSE, uniquely arising from the interplay between surface reconstruction and non-Hermitian physics.

### 16. Possible novel phenomena of boundary-reconstructed photonic crystals

Boundary reconstruction in photonic crystal slabs, when combined with intrinsic non-Hermitian effects like far-field radiation and optical loss/gain, can lead to novel phenomena and functionalities beyond those of conventional non-Hermitian optical systems. Here are some possible novel phenomena:

(1) Boundary reconstruction modifies the geometry and symmetry of the photonic crystal slab's edge or surface. This can result in the creation of localized edge or interface modes that are uniquely sensitive to non-Hermitian effects such as radiation and gain/loss asymmetries.

(2) The reconstructed boundary can break reciprocity in photon hopping or scattering, amplifying the effects of intrinsic non-Hermitian features.

(3) Reconstructed boundaries can couple localized states (e.g., surface modes) with far-

field radiation or leaky modes, producing new quasi-bound states with distinct spectral properties.

(4) Asymmetric coupling between reconstructed boundary modes and bulk photonic states could induce boundary-localized states via the NHSE, leading to photon accumulation near the reconstructed boundary.

(5) Boundary reconstruction could create new exceptional points by coupling surface or interface states with non-Hermitian perturbations like gain/loss or radiation.

(6)The reconstructed boundary could induce NHSE-like effects, where light accumulates at specific regions of the boundary due to asymmetric couplings. This is distinct from conventional bulk NHSE, as it occurs at the boundary level and leverages photonic-specific non-Hermitian effects.

(7) The reconstructed boundary might interact with intrinsic non-Hermitian effects to create novel topological phases or edge states.

Boundary reconstruction in photonic crystal slabs can synergize with intrinsic non-Hermitian effects to create phenomena that go beyond those in conventional non-Hermitian systems. These include the emergence of surface NHSE, reconstruction-induced EPs, asymmetric radiation, and enhanced topological effects. A surface-reconstructed photonic crystal could be realized by overlaying a mask on the surface of a conventional photonic crystal. By altering the mask's shape, different surface-reconstructed photonic crystals can be simulated and experimentally achieved, allowing for precise control of surface reconstruction. This approach paves the way for new possibilities in photonic device design, potentially unlocking innovative functionalities and applications.

Interestingly, the photoelectric detection process inherently establishes an open boundary condition. The escape of photoelectrons from the material resembles the mechanism of far-field radiation and photon loss. Drawing comparisons between far-field radiation or optical gain/loss and electronic systems reveals intriguing phenomena, as below:

(1) Surface reconstruction alters the electronic band structure near the surface, potentially creating new surface states or modifying existing ones. These states can directly affect the initial conditions of photoelectrons emitted during the photoelectric process.

(2) Surface reconstructions in Weyl semimetals may introduce additional non-Hermitian characteristics due to coupling with open boundaries and potential losses. This can impact the lifetime and dynamics of surface-bound electronic states, influencing the energy and

momentum distribution of emitted photoelectrons.

(3) If the Weyl semimetal exhibits topological surface states, reconstruction could modify these states, potentially introducing asymmetry or anisotropy in the photoelectron emission. This could provide a unique signature useful in photoelectric detection.

---

[1] J. Gaudet *et al.*, Weyl-mediated helical magnetism in NdAlSi. Nat. Mater. **20**, 1650 (2021).

[2] J. F. Wang *et al.*, NdAlSi: A magnetic Weyl semimetal candidate with rich magnetic phases and atypical transport properties. Phys. Rev. B **105**, 144435 (2022).

[3] D. E. Laughlin and K. Hono, Crystal Structures of Metallic Elements and Compounds. Physical Metallurgy (Fifth Edition) Elsevier, (2015). https://doi.org/10.1016/C2010-0-65716-6

[4] I. Belopolski *et al.*, Criteria for Directly Detecting Topological Fermi Arcs in Weyl Semimetals. Phys. Rev. Lett. **116**, 066802 (2016).

[5] C. Li *et al.*, Emergence of Weyl fermions by ferrimagnetism in a noncentrosymmetric magnetic Weyl semimetal. Nat. Commun. **14**, 7185 (2023).

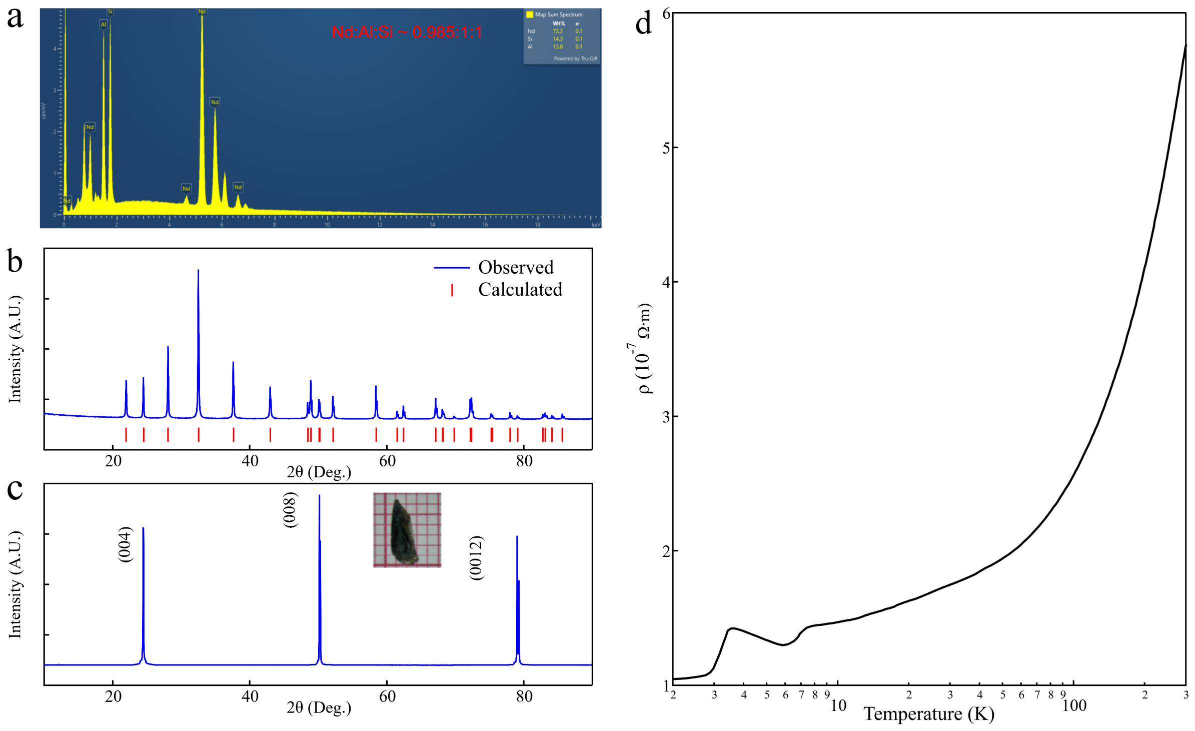


FIG. S1. **Crystal characterizations of NdAlSi.** (a) Typical energy dispersive x-ray (EDX) spectra of NdAlSi single crystal. (b) Rietveld refinement of the powder XRD patterns of NdAlSi. (c) Single crystal XRD peaks of NdAlSi. The inset shows an optical image of an as grown single crystal of NdAlSi. (d) Temperature-dependent resistivity $\rho(T)$ measured under zero field with I//a.

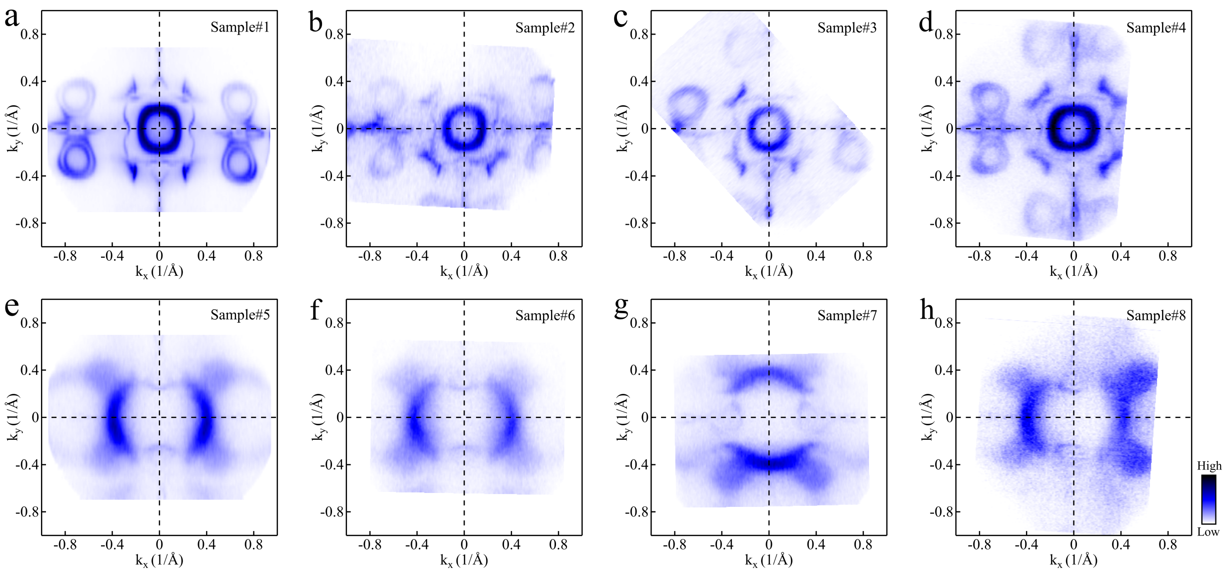


FIG. S2. **Fermi surface of NdAlSi measured from 8 different samples with photon energy of 41 eV.** The measured Fermi surfaces of (a-d) correspond to surface 1 marked in Fig. 1e in the main text, and the measured Fermi surfaces of (e-h) correspond to surface 2 marked in Fig. 1f in the main text.

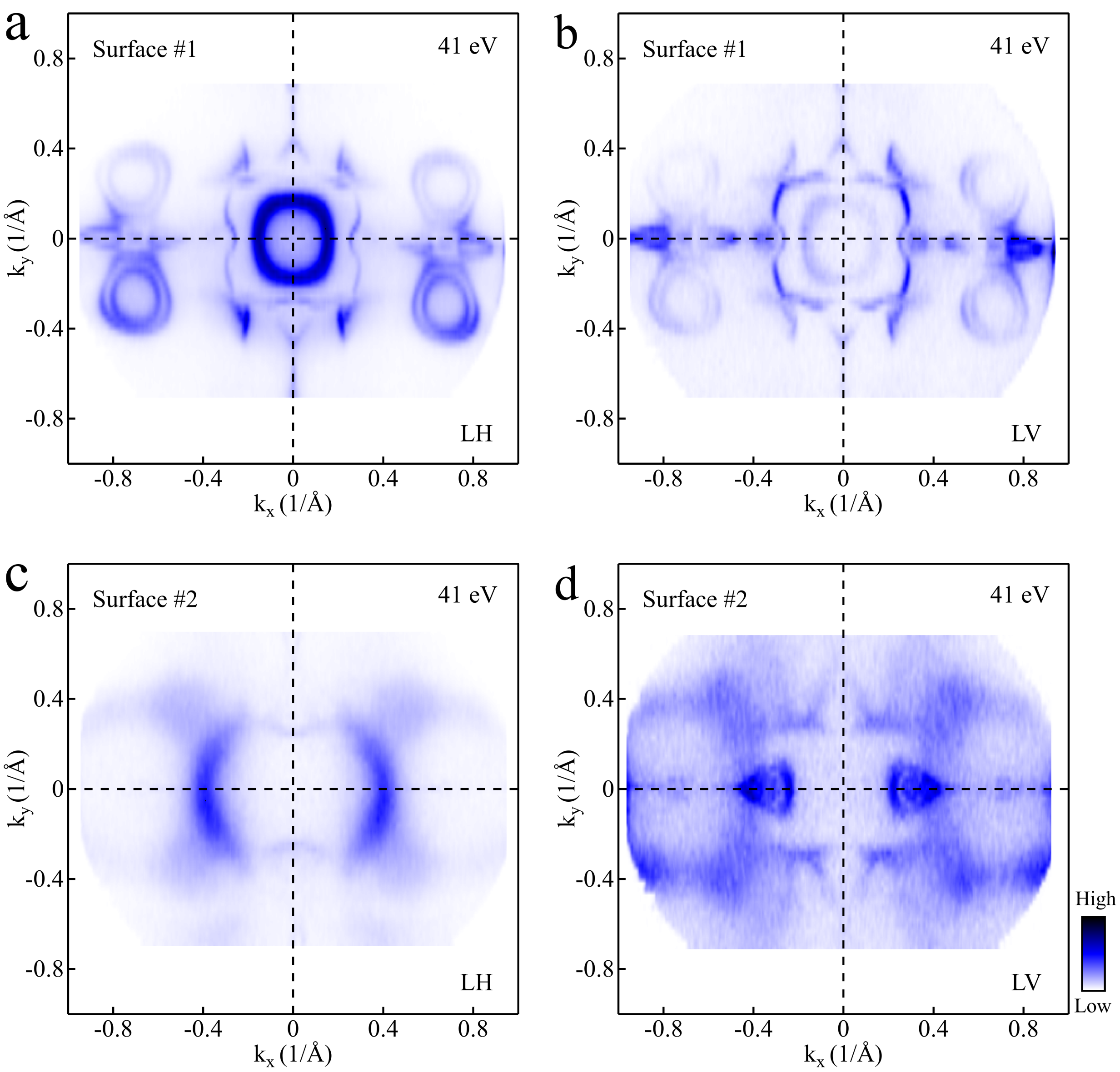


FIG. S3. **Fermi surfaces of NdAlSi measured under different polarizations.** (a-d) The Fermi surfaces of NdAlSi measured on surface 1 (a-b) and surface 2 (c-d) with photon energy of 41 eV under LH (a, c) and LV (b, d) polarizations.

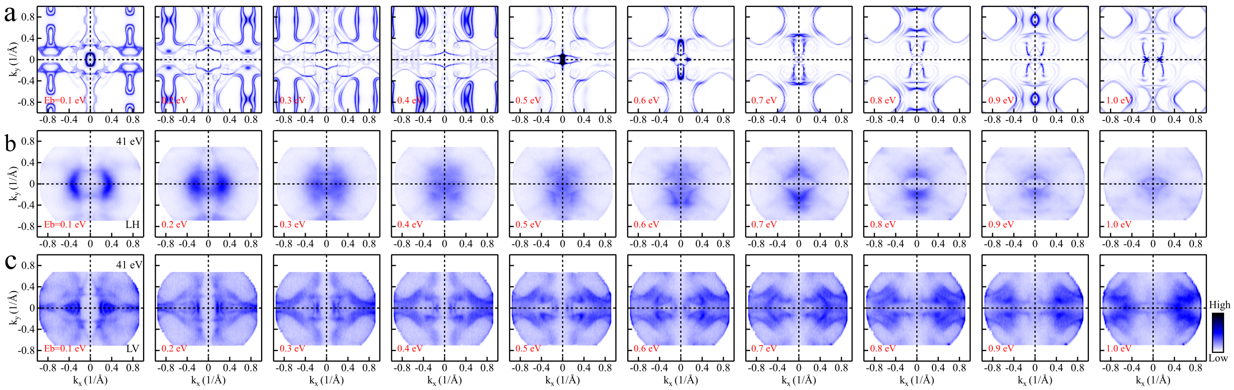


FIG. S4. **Measured and calculated constant energy contours of NdAlSi.** (a) The surface projected DFT CEC calculations of the Al terminal surface cleavage at the Al-Nd layer without considering the surface reconstruction. (b-c) The measured CECs on surface 2 with photon energy of 41 eV under LH (b) and LV (c) polarizations.

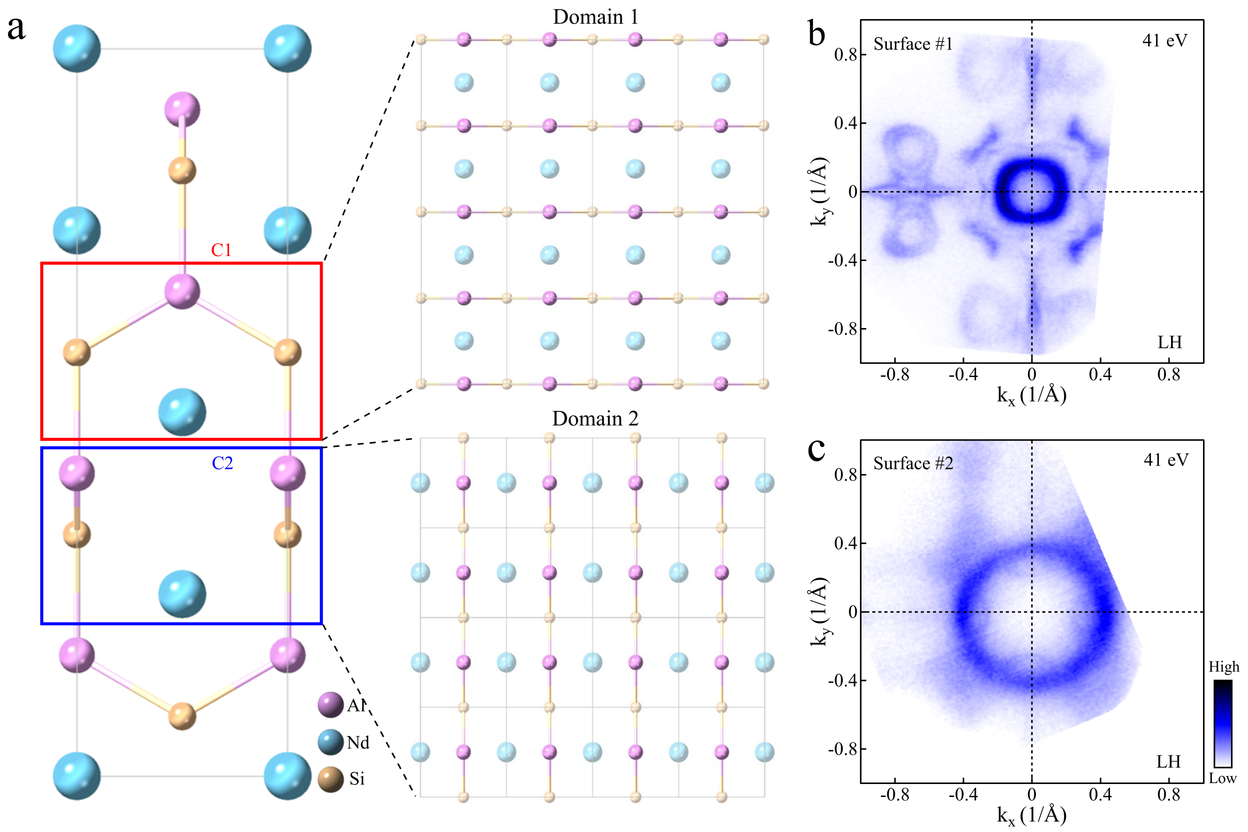


FIG. S5. **The twinned Fermi surface of NdAlSi.** (a) Lattice structure diagram of the cleavage plane of NdAlSi. (b-c) The Fermi surfaces of surface 1 (b) and surface 2 (c) measured on twinned surface structure of NdAlSi with photon energy of 41 eV under LH polarization.

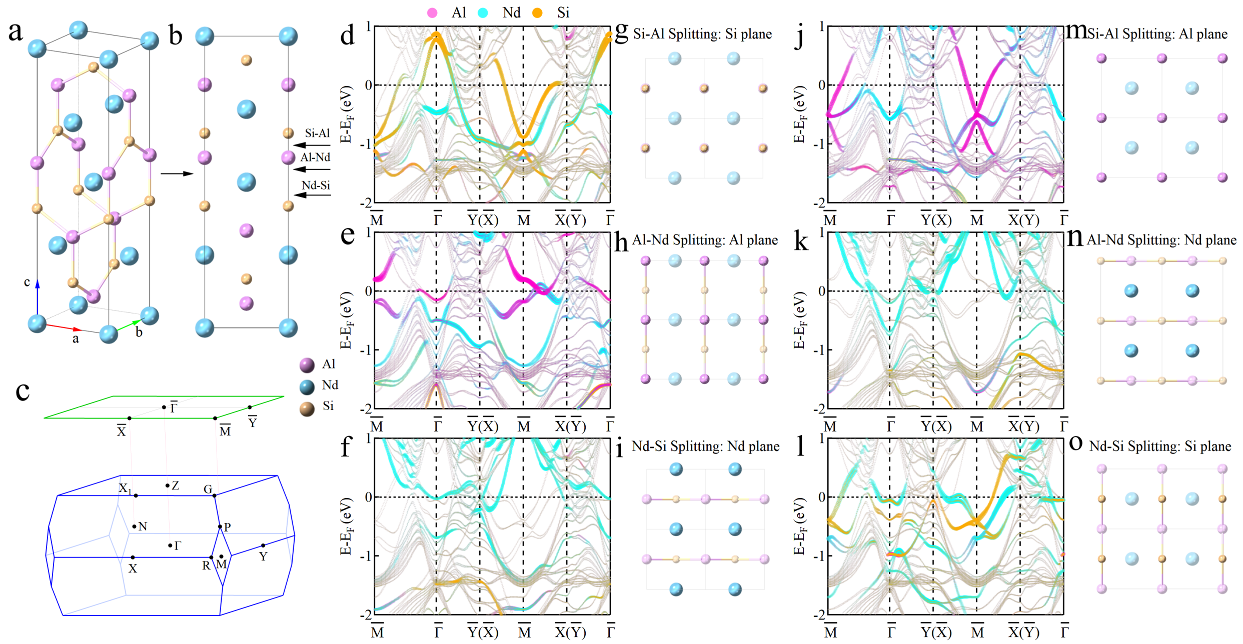


FIG. S6. **Crystal structure and termination surface dependent band structure calculations for NdAlSi.** (a) The crystal structure of NdAlSi with the space group $I4_1md$ (no. 109). (b) The side view of (a). (c) The 3D Brillouin zone (BZ) of the original unit cell of NdAlSi, and the corresponding two-dimensional BZ projected on the (001) plane (green lines) in the pristine phase in (a). (d-f, j-l) The surface projected DFT calculated band dispersion along $\overline{M}-\overline{\Gamma}-\overline{Y}(\overline{X})-\overline{M}-\overline{X}(\overline{Y})-\overline{\Gamma}$ directions and their atom nature on the terminal surface of Si atom (d) cleavage at the Si-Al layer, Al atom (e) cleavage at the Al-Nd layer, Nd atom (f) cleavage at the Nd-Si layer, Al atom (j) cleavage at the Si-Al layer, Nd atom (k) cleavage at the Al-Nd layer and Si atom (l) cleavage at the Nd-Si layer. Where the pink bands represent the orbital from the Al atom, the cyan bands represent the orbital from the Nd atom and the orange bands represent the orbital from the Si atom. The spectral intensity is illustrated by the size and transparency of the markers. The $\overline{X}$ and $\overline{Y}$ here depend on the definition of the $a$ and $b$ axes of the lattice structure. (g-i, m-o) The termination surface corresponding to the surface projected DFT calculations of (d-f, j-l). Bright colors represent the topmost atoms, and light colors represent the lower atoms.

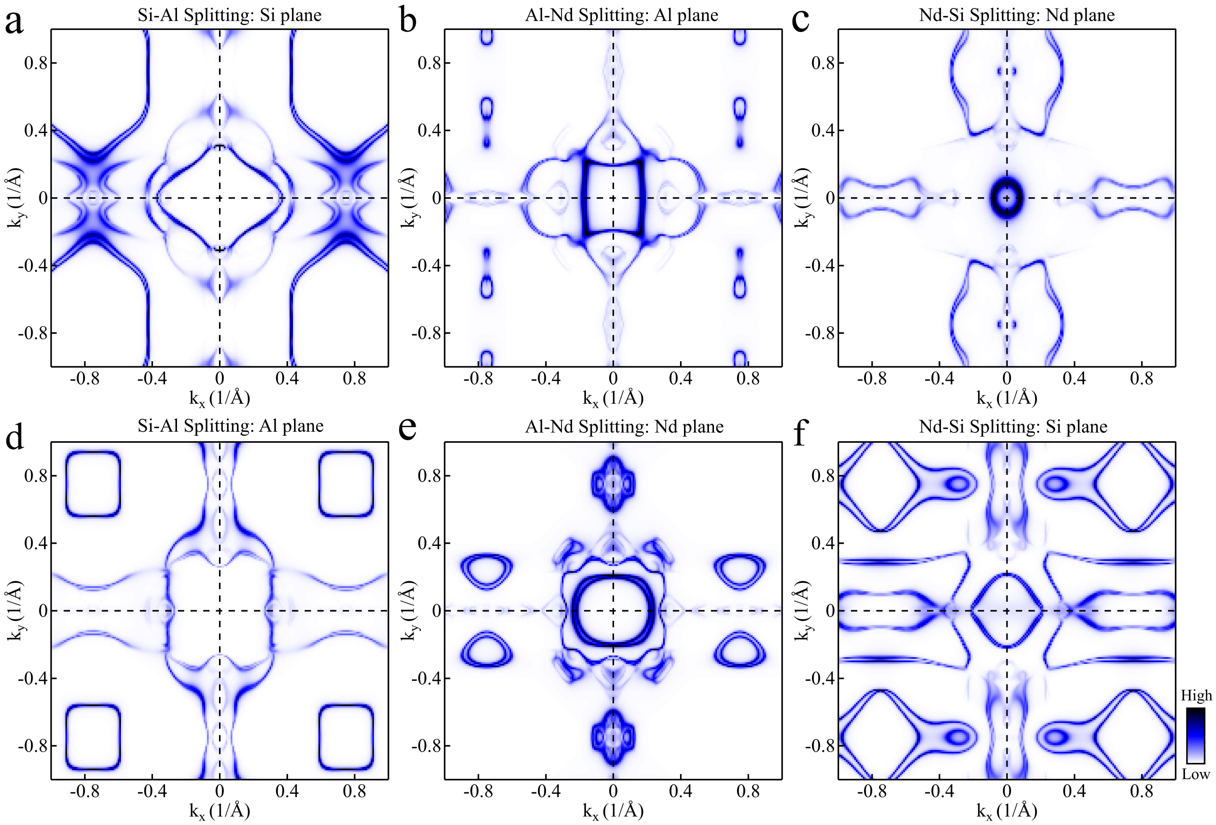


FIG. S7. **Surface projected DFT Fermi surfaces of NdAlSi.** (a-f) The surface projected DFT calculated Fermi surfaces on the Si terminated surface (a) and Al terminated surface (d) cleaved at the Si-Al layer, Al (b) and Nd (e) terminated surfaces cleaved at the Al-Nd layer, Nd (e) and Si (f) terminated surfaces cleaved at the Nd-Si layer.

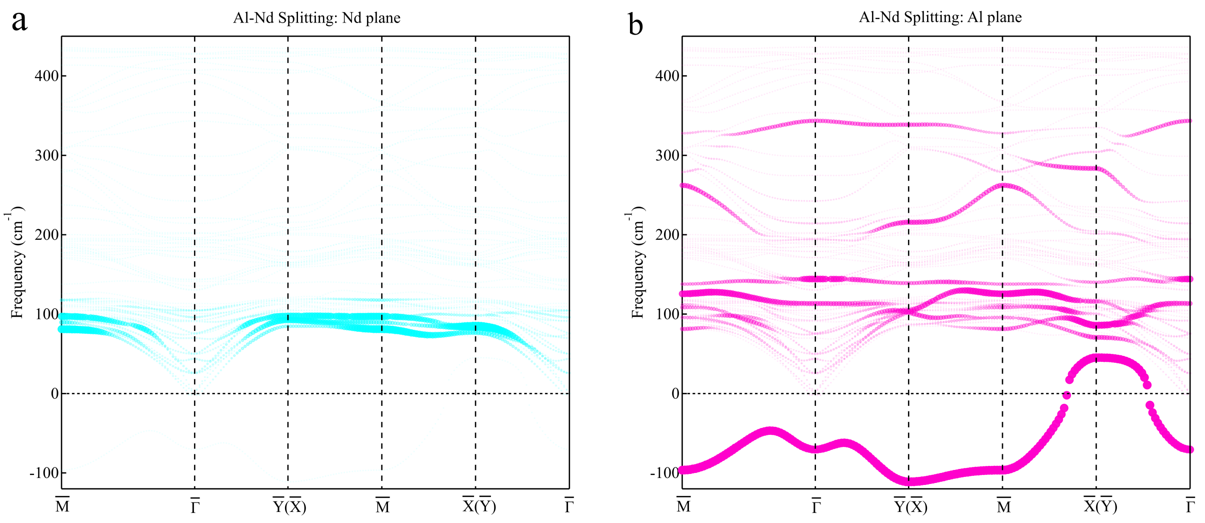


FIG. S8. **The surface projected phonon spectrum calculations of NdAlSi.** (a-b) The surface projected phonon spectrum calculations on the Nd (a) and Al (b) terminated surfaces at the Al-Nd layer. The spectral intensity is denoted by the size and transparency of the markers. The higher the intensity, the greater the proportion of surface projection.

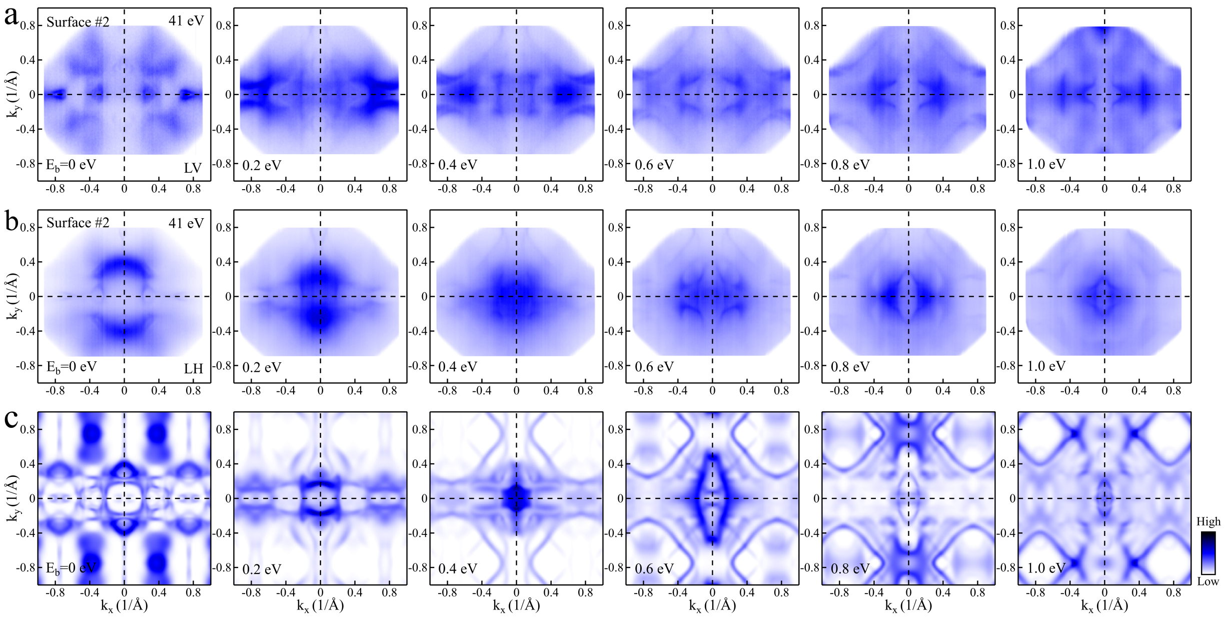


FIG. S9. **Comparison of the constant energy contours at different binding energies.** (a-b) Fermi surfaces and constant energy contours of NdAlSi measured on surface 2 with photon energy of 41 eV under LV (a) and LH (b) polarizations. (c) The surface projected DFT Fermi surface and constant energy contours calculated for NdAlSi on the Al terminated surface cleaved at the Al-Nd layer with inclusion of surface reconstruction.

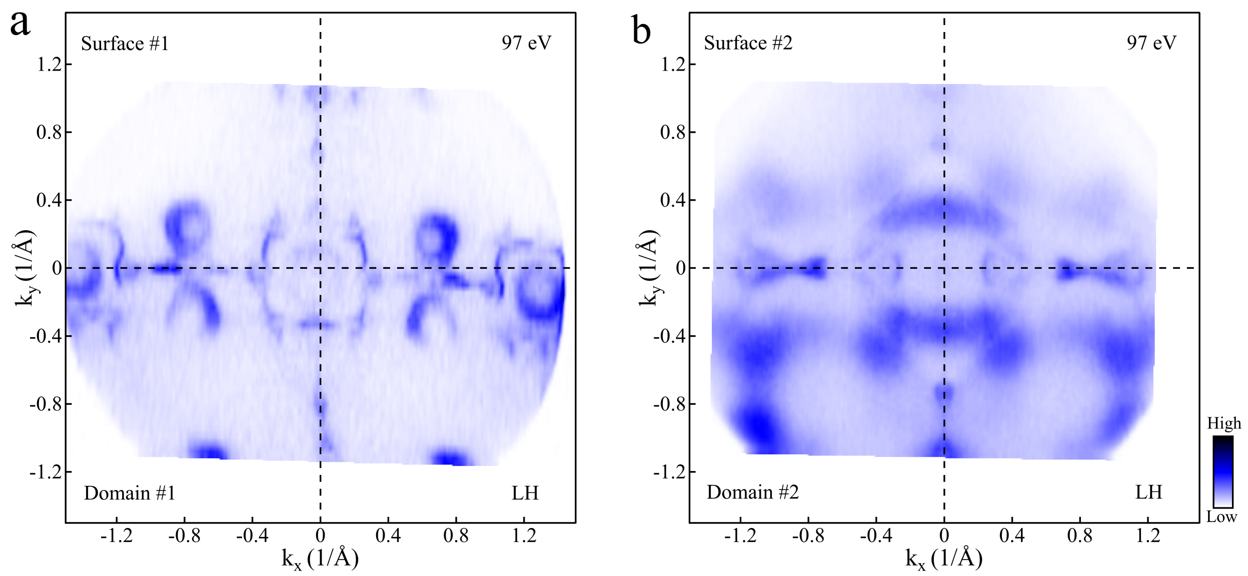


FIG. S10. **Fermi surfaces of NdAlSi measured with photon energy of 97 eV.** (a-b) Fermi surface maps of NdAlSi measured on surface 1 (a) and surface 2 (b) with photon energy of 97 eV.

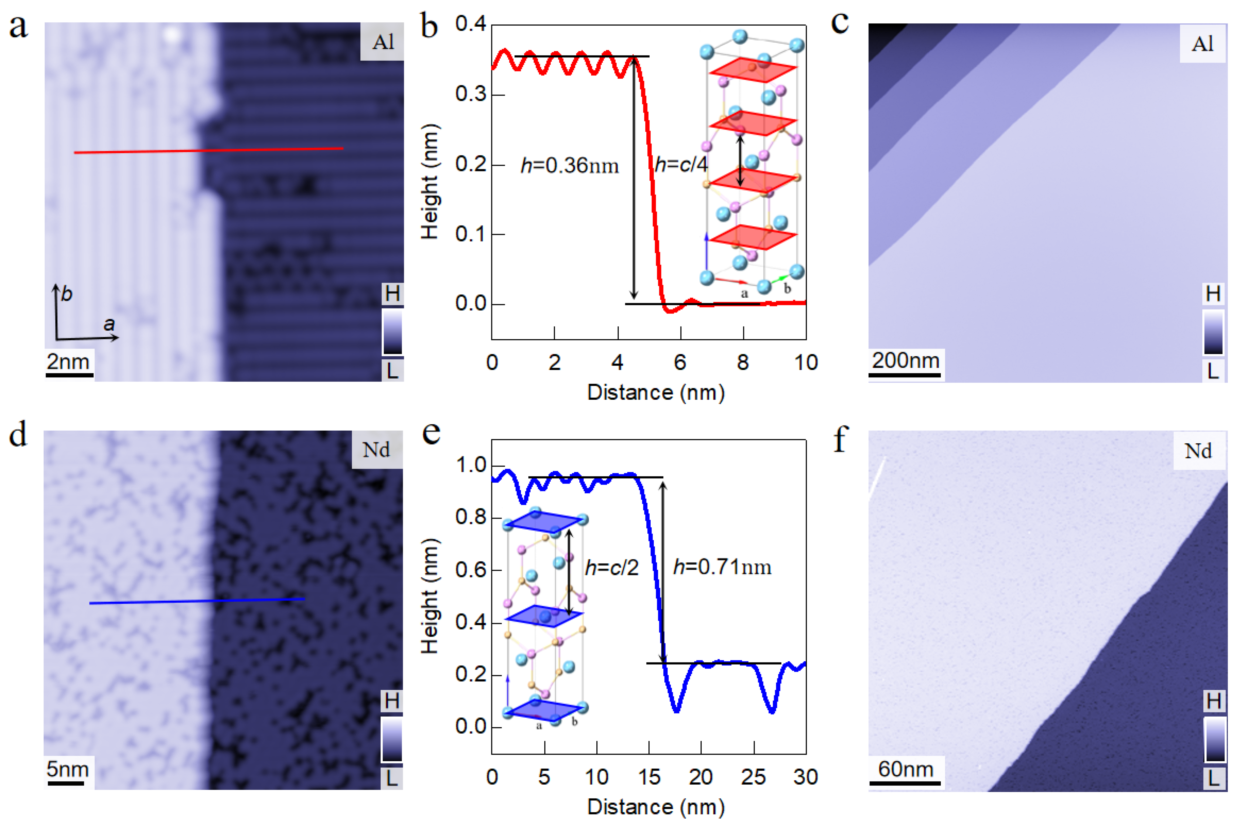


FIG. S11. **The STM measurements of NdAlSi.** (a) STM image of NdAlSi on the Al terminated surface at the Al-Nd layer measured at 0.3 K. Line profile along the red line is shows in (b). (c) STM image on a larger scale of NdAlSi on the Al terminated surface at the Al-Nd layer measured at 4.7 K. (d-f) Similar measurements as in (a-c) but measured on the Nd terminated surface at 4.7 K. Scan conditions: (a) -1.5 V, 0.5 nA, (c) -1.0 V, 0.1 nA, (d) -0.5 V, 0.5 nA, (f) 0.5 V, 0.5 nA. Negative bias implies filled states and vice versa.

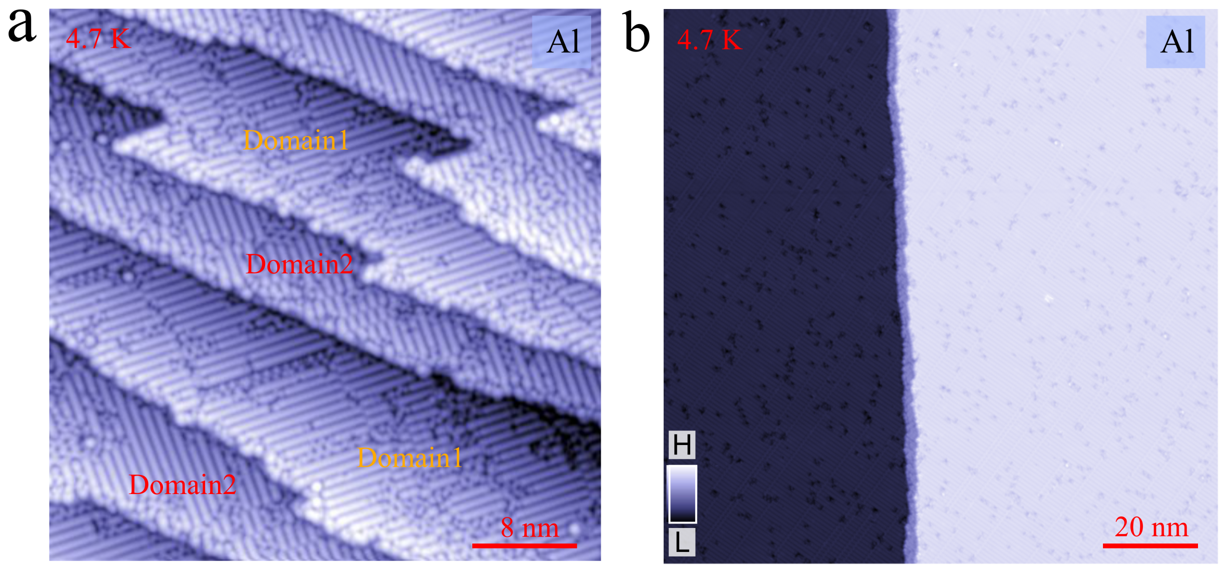


FIG. S12. STM images of NdAlSi measured on uneven (a) and flat (b) area of Al atom terminated surfaces cleavage at the Al-Nd layer. Scan conditions: (a) -1 V, 0.5 nA, (b) 0.3 V, 0.6 nA.

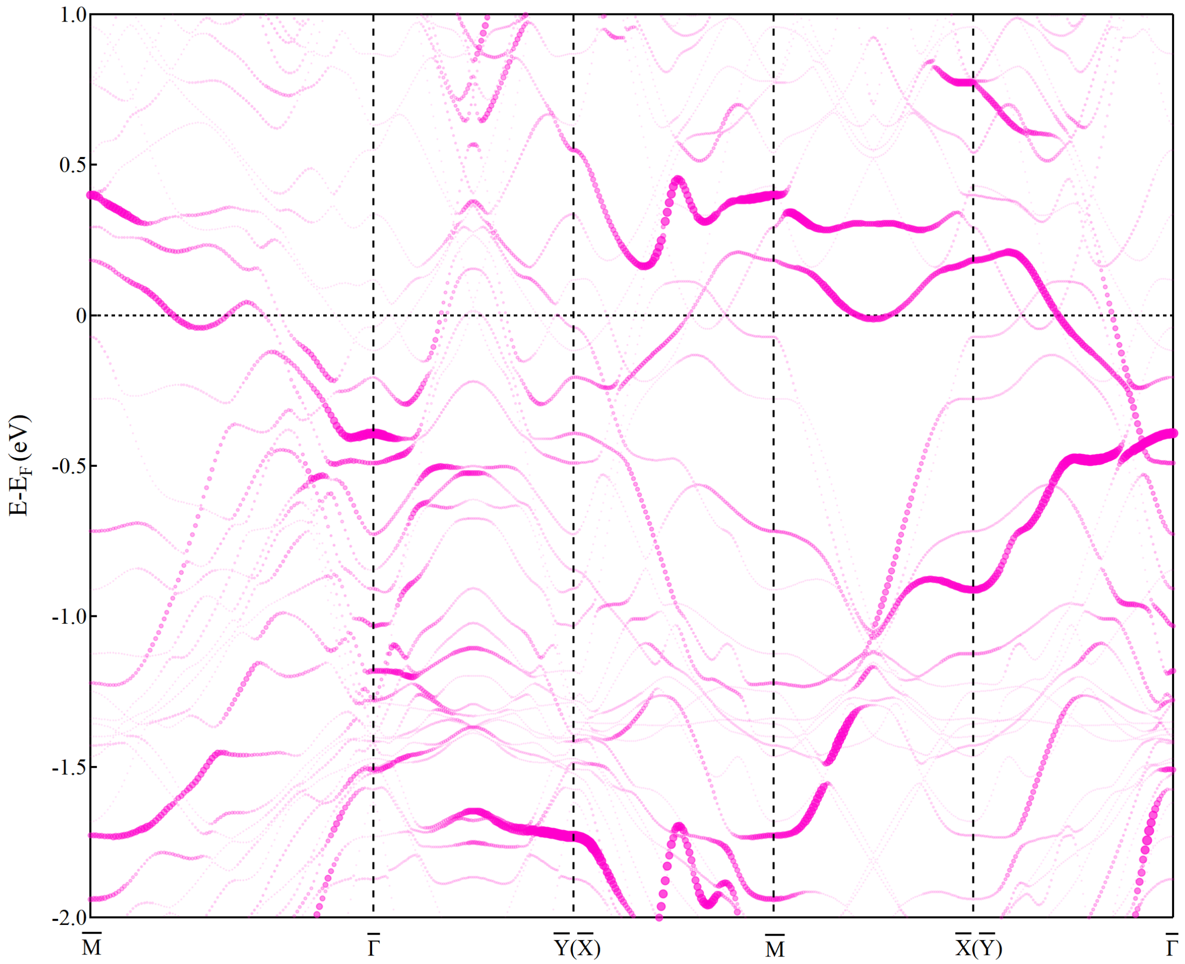


FIG. S13. **Surface projected DFT band calculations of NdAlSi with surface reconstruction included.** The surface projected DFT calculated band dispersion along high-symmetry directions across the BZ of the Al terminated surface cleaved at the Al-Nd layer with inclusion of surface reconstruction.

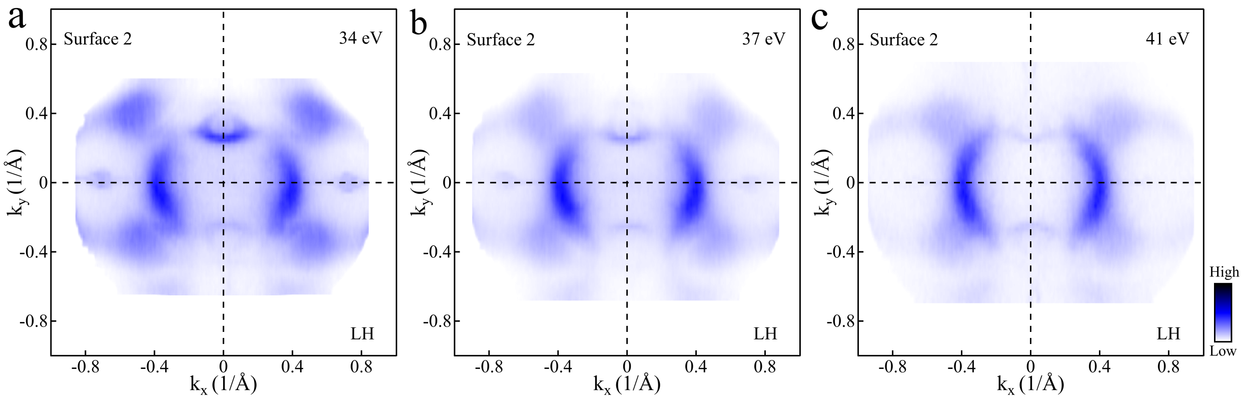


FIG. S14. **Photon energy dependent Fermi surfaces of NdAlSi measured on surface 2.** (a-c) Fermi surface of NdAlSi measured on surface 2 with the photon energy of 34 eV (a), 37 eV (b) and 41 eV (c) under LH polarization.

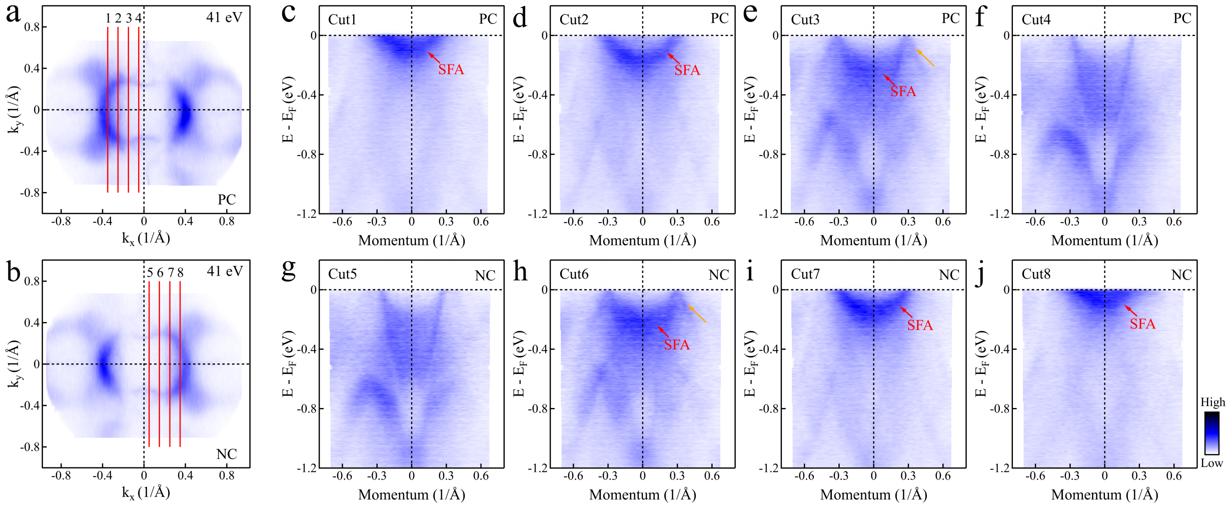


FIG. S15. **Fermi surfaces and band dispersions of NdAlSi.** (a-b) Fermi surface of NdAlSi measured on surface 2 with photon energy of 41 eV under PC and NC polarizations. (c-f) Band dispersions measured on surface 2 along Cut1-Cut4 with photon energy of 41 eV under PC polarization. (g-j) Band dispersions measured on surface 2 along Cut5-Cut8 with photon energy of 41 eV under NC polarization.

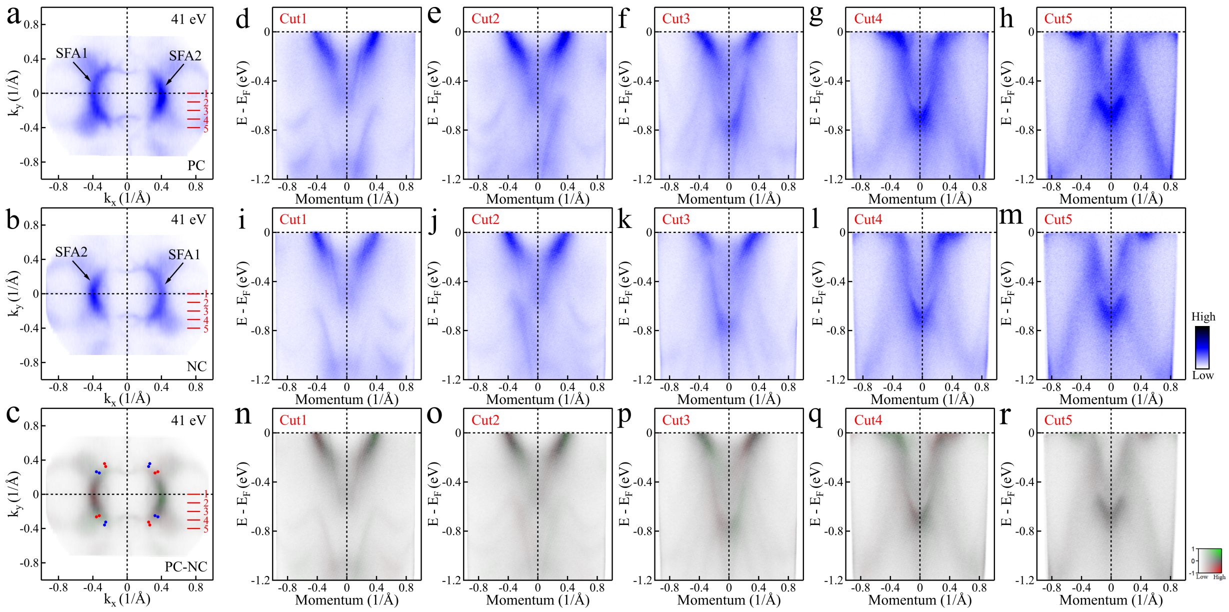

FIG. S16. **The CD measurements on surface 2.** (a-b) The circular polarization dependent Fermi surfaces on surface 2 measured with photon energy of 41 eV under PC (a) and NC (b) polarization. (c) The CD results of Fermi surface. (d-m) The circular polarization dependent band structures measured with photon energy of 41 eV under PC (d-h) and NC (i-m) polarization. The location of band cuts are defined in (a-b). (n-r) The CD results of band structures corresponding to (d-m). The red dots representing nodes with chiralities +1 and blue dots representing -1.

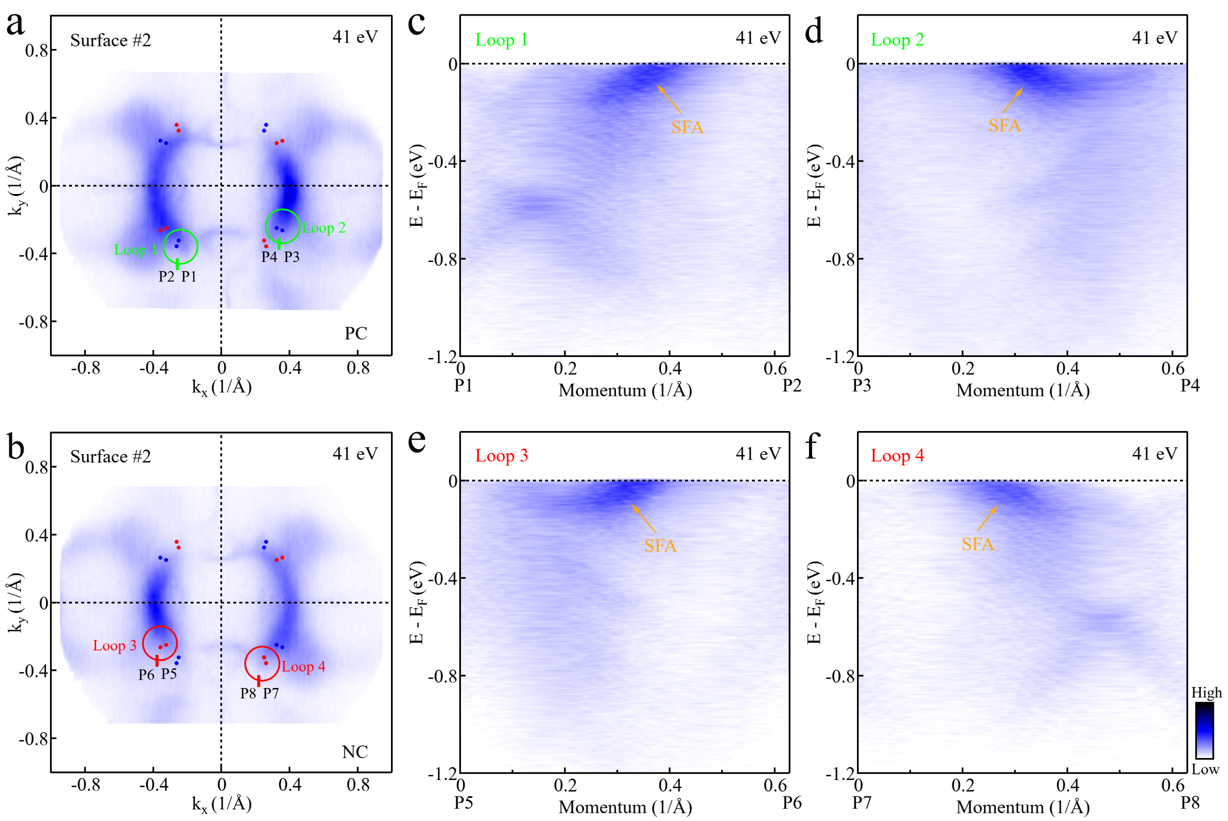


FIG. S17. **The surface projected chiral charge of arc features.** (a) The circular polarization dependent Fermi surfaces on surface 2 measured with photon energy of 41 eV under PC (a) and NC (b) polarization. (c-f) Band dispersions along Loop 1 (c), Loop 2 (d), Loop 3 (e) and Loop 4 (f). The red dots representing nodes with chiralities +1 and blue dots representing -1.

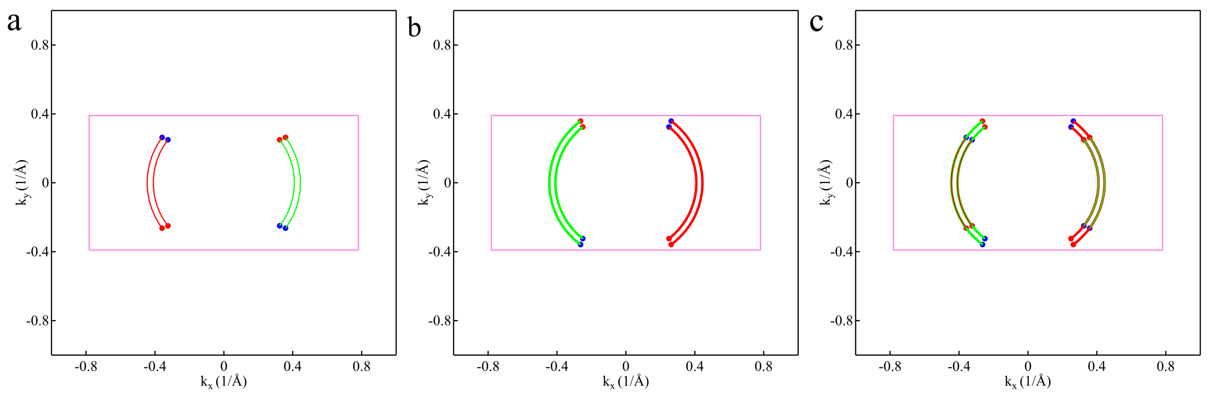


FIG. S18. **Fermi arcs of surface 2.** (a) Fermi arcs connecting the inner Weyl nodes. (b) Fermi arcs connecting the outer Weyl nodes. (c) Schematic of Weyl node connections of surface 2. Pink lines are the first BZ boundary at the $k_z = 0\ \pi/c$ plane with considering the surface reconstruction.

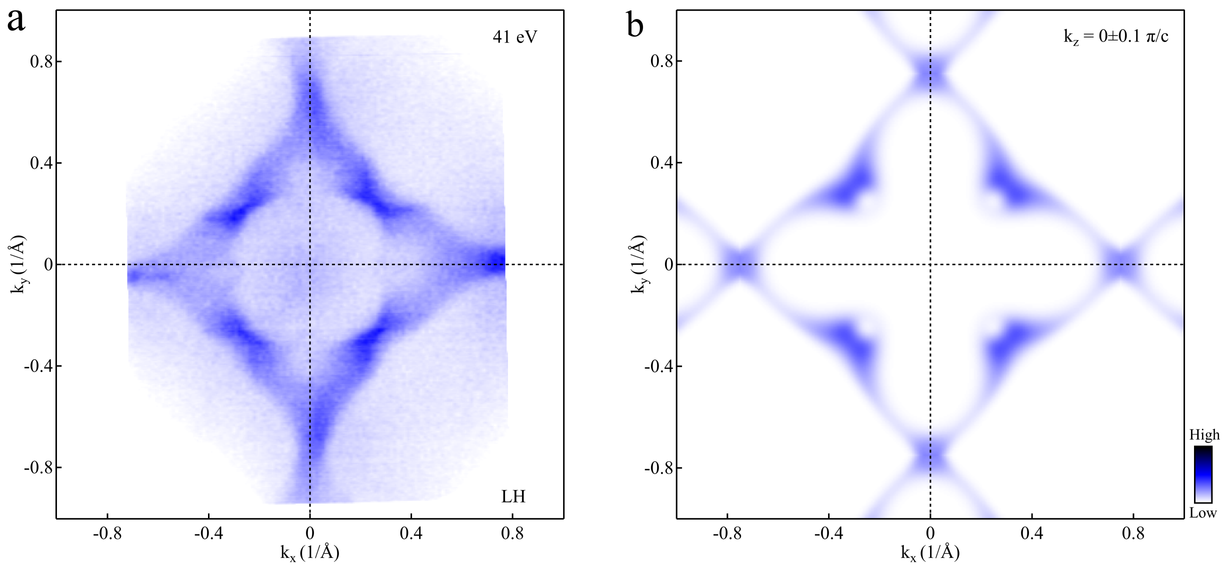


FIG. S19. **Bulk Fermi surface of NdAlSi.** (a) Bulk Fermi surface of NdAlSi measured with photon energy of 41 eV under LH polarization. (b) The DFT calculated bulk Fermi surface at the $k_z = 0\ \pi/c$ plane, integrated $0\pm0.1\ \pi/c$ over the BZ along the $k_z$ direction.

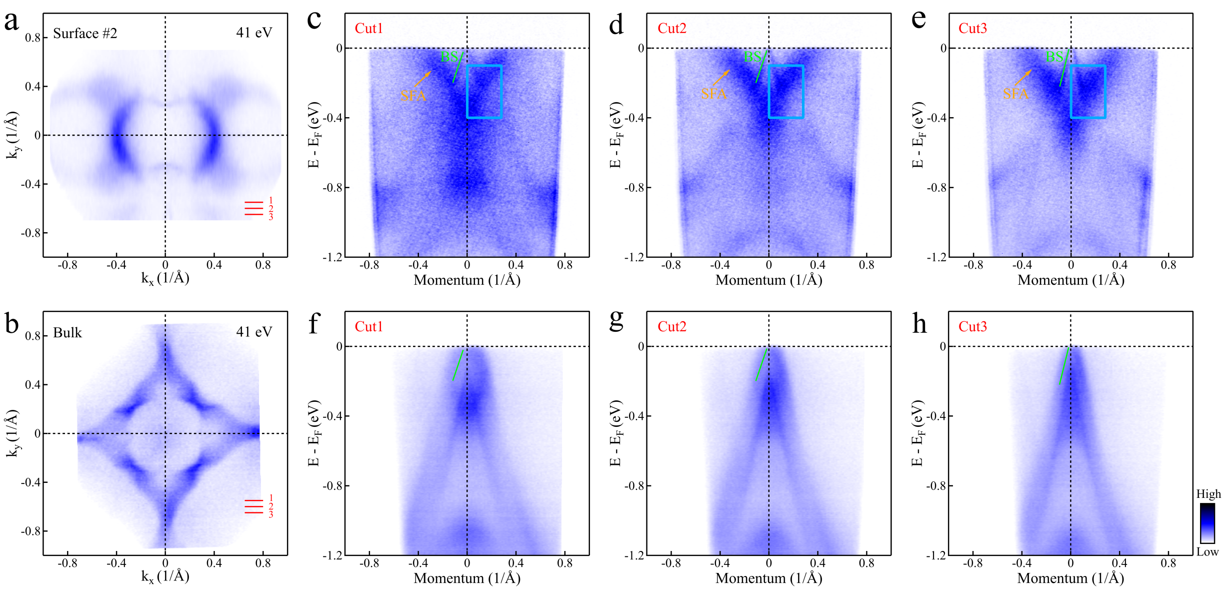


FIG. S20. **Comparison of surface states and bulk states.** (a-b) Fermi surface of surface 2 (a) and bulk state (b) in NdAlSi measured with photon energy of 41 eV under LH polarization. (c-e) The band dispersions measured on surface 2 along Cut1-Cut3 with photon energy of 41 eV under LH polarization. (f-h) The bulk band dispersions along Cut1-Cut3 with photon energy of 41 eV under LH polarization. Green lines in (c-e) mark the band dispersions in corresponding panels. The same lines are replotted in (f-h) for comparison. The area surrounded by the cyan lines represent the bands hybrid area.

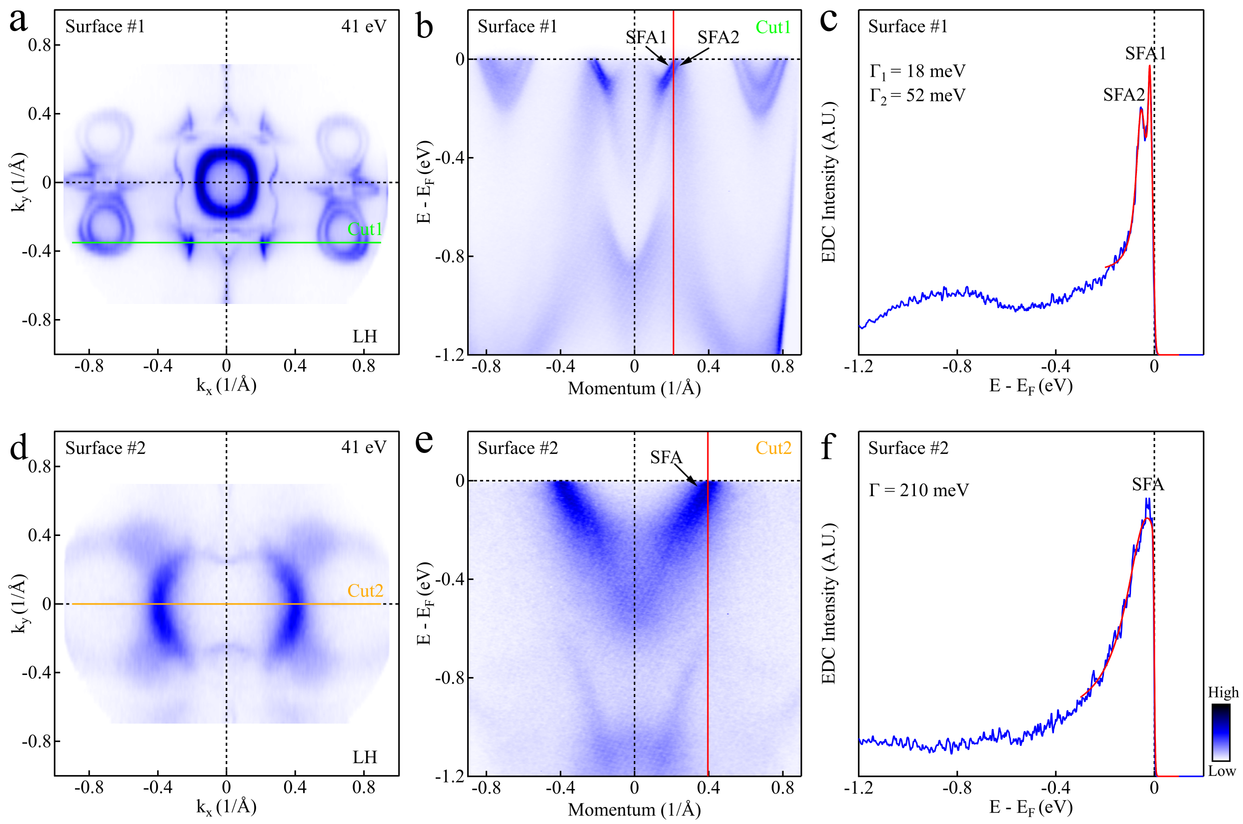


FIG. S21. **Quasiparticle scattering rate of SFA.** (a) Fermi surface of NdAlSi measured with photon energy of 41 eV under LH polarization on Nd-terminated surface. (b) Band structure measured along Cut1 in (a). (c) Energy distribution curve (EDC) extracted along red line in (b). The red curve in (c) is the fitted EDC considering two Lorentz peaks, which gives a scattering rate of 18 meV for SFA1 and 52 meV for SFA2. (d-f) The similar measurements and analysis as (a-c). The scattering rate of SFA obtained by fitting EDC is 210 meV.
